# Genetic Spectrum, Family History and Diagnostic Yield of Targeted Genetic Testing in Early-Onset Alzheimer’s Disease and Frontotemporal Dementia: A Serbian Memory Clinic

**DOI:** 10.3390/diseases14100359

**Published:** 2026-09-29

**Authors:** Gorana Mandić-Stojmenović, Vladimir Kostić, Ana Marjanović, Ivana Novaković, Predrag Aleksić, Tanja Stojković, Elka Stefanova

**Affiliations:** 1 Neurology Clinic, University Clinical Centre of Serbia, 11000 Belgrade, Serbiaana.marjanovic@yahoo.com (A.M.); steela21@gmail.com (E.S.); 2 Faculty of Medicine, University of Belgrade, 11000 Belgrade, Serbia; tetaana61@yahoo.com

**Keywords:** early-onset dementia, Alzheimer’s disease, frontotemporal dementia, family history, genetic testing, pathogenic variants

## Abstract

Background/Objectives: Early-onset dementia (EOD), defined as symptom onset before 65 years of age, is a clinically and genetically heterogeneous group. Alzheimer’s disease (AD) and frontotemporal dementia (FTD) are its most common neurodegenerative causes. Methods: We investigated the family history, genetic spectrum, and diagnostic yield of targeted genetic testing in a consecutive Serbian cohort of 130 patients with early-onset AD (EOAD) and 78 with early-onset FTD (EOFTD). Results: Approximately one-third of patients had a positive family history of dementia or another neurodegenerative disorder. Pathogenic or likely pathogenic variants were identified in 3.8% of patients with EOAD, whereas pathogenic or likely pathogenic variants or pathogenic repeat expansions were identified in 9.0% of patients with EOFTD. In EOAD, the diagnostic yield increased to 9.5% in those with a positive family history and, in an exploratory subgroup analysis, was 33.3% in those with symptom onset before 51 years. In EOFTD, the diagnostic yield was 25.0% among those with a positive family history. *PSEN1* and *APP* were the only disease-causing genes identified in EOAD, whereas *C9orf72* repeat expansion was the predominant genetic cause of EOFTD, with additional pathogenic variants identified in *GRN* and *VCP*. Conclusions: These findings support the clinical value of targeted genetic testing guided by family history, age at onset, and clinical phenotype in patients with EOD. Genetically unresolved familial cases may reflect incomplete assessment of known genetic causes, including genes and variant classes not covered by the targeted testing strategy used in this study.

## Simple Summary

Early-onset dementia refers to dementia with symptom onset before the age of 65 years and can be difficult to diagnose because younger patients often present with behavioral, psychiatric, or cognitive symptoms. Alzheimer’s disease and frontotemporal dementia are the most common neurodegenerative causes of early-onset dementia, and both have an important genetic component. This study investigated the genetic characteristics and the molecular diagnostic yield of genetic testing in Serbian patients with early-onset dementia, focusing on the relationship between genetic findings, family history, and clinical features. Genetic testing identified disease-causing variants in a subset of patients, particularly among those with a strong family history or very early disease onset. These findings highlight the potential contribution of genetic testing to the etiological characterization of selected patients with early-onset dementia and to providing relevant genetic information for patients and their families.

## 1. Introduction

Early-onset dementia (EOD), conventionally defined as dementia with symptom onset before the age of 65 years [1], accounts for approximately 5% of all dementia cases, with an estimated global prevalence of 119 cases per 100,000 individuals aged 30–64 years [2]. The global age-standardized incidence has been reported at approximately 11 cases per 100,000 person-years in this age group, with substantial geographical variation across studies [3]. This variation may reflect differences in study methodology, case ascertainment, population characteristics, and the availability of epidemiological data across geographical regions [2,3]. Despite substantial advances in neuroimaging and fluid biomarkers, establishing an early and accurate diagnosis of EOD remains challenging, even in specialized memory clinics [1,4]. The marked clinical heterogeneity of EOD, together with younger age at symptom onset and the high frequency of atypical clinical presentations, including behavioral, psychiatric, and non-amnestic cognitive phenotypes, contributes to delayed diagnosis compared with late-onset dementia [5,6].

Alzheimer’s disease (AD) and frontotemporal dementia (FTD) are the two most common degenerative causes of EOD. Their early-onset forms are referred to as early-onset Alzheimer’s disease (EOAD) and early-onset frontotemporal dementia (EOFTD), respectively. Genetic factors play a more prominent role in EOAD than in late-onset AD (LOAD), with heritability estimates ranging from 92% to 100% [7]. Pathogenic variants in the amyloid precursor protein (*APP*), presenilin 1 (*PSEN1*), and presenilin 2 (*PSEN2*) genes are well-established causes of autosomal dominant EOAD [8,9,10], but account for only 5–10% of all EOAD cases [7]. These genes converge on amyloid-β (Aβ) biology: *APP* encodes the amyloid precursor protein, whereas presenilins form the catalytic core of the γ-secretase complex involved in *APP* processing and Aβ generation. Aβ accumulation may, in turn, contribute to other pathological processes in AD, including tau pathology [11]. Metabolic factors, including glycemic variability and hypoglycemia, have also been discussed in the broader context of neurodegenerative disorders [12]. The apolipoprotein E (*APOE*) ε4 allele is the strongest common genetic risk factor for sporadic LOAD and is also associated with an increased risk of EOAD [13]. Nevertheless, despite advances in next-generation sequencing (NGS), which have expanded the genetic landscape of EOAD, the genetic basis of many familial cases remains unresolved [7,13]. More than one-third of patients with FTD have a family history of dementia or related neurodegenerative disorders [14]. Most monogenic FTD cases are associated with *C9orf72* repeat expansions and pathogenic variants in progranulin (*GRN*) and microtubule-associated protein tau (*MAPT*), although their relative frequencies vary across populations, with *C9orf72* being the most common genetic cause worldwide [15]. At the molecular level, genetic FTD is highly heterogeneous, with disease-associated genes implicating multiple cellular pathways, including ribonucleic acid (RNA) metabolism, protein degradation and clearance, and lysosomal/endosomal function [16]. Pathogenic variants in several other genes, including *TBK1*, *VCP*, *CHMP2B*, *TARDBP*, and *FUS*, have been associated with familial FTD, but collectively account for less than 5% of all cases [15]. Although both EOAD and FTD have a strong genetic component, autosomal dominant inheritance is identified only in a minority of EOD cases. In a clinically comparable EOD cohort, an autosomal dominant inheritance pattern was reported in 14.2% of patients with EOAD and 13.4% of patients with EOFTD, with known pathogenic variants explaining only 11.8% and 45.5% of these cases, respectively [17].

Characterization of EOD cohorts from different geographical populations is important for broadening our understanding of the genetic spectrum of EOAD and EOFTD and for informing genetic testing in different clinical settings. Building on previous Serbian genetic studies, the present study extends previous reports through additional phenotype-directed genetic testing and a focused analysis of early-onset disease [18,19,20].

The aim of this study was to investigate the genetic spectrum and diagnostic yield of targeted genetic testing, defined as the proportion of tested patients with an identified pathogenic or likely pathogenic variant or pathogenic repeat expansion, in a consecutive cohort of patients with EOAD and EOFTD, and to examine its relationship with family history and clinical characteristics.

## 2. Materials and Methods

### 2.1. Study Population and Diagnostic Assessment

**Figure 1:**
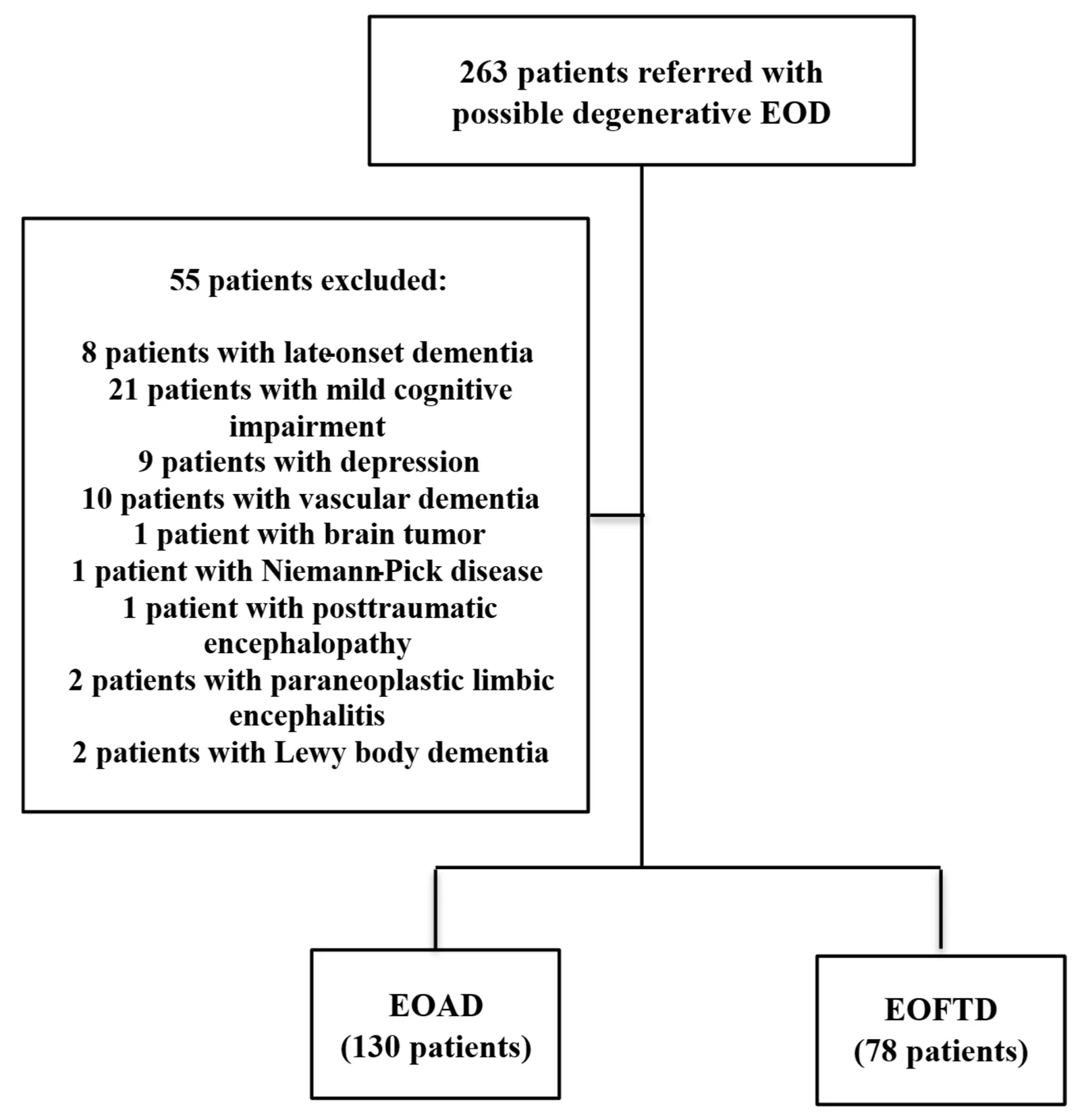
Flowchart of patient selection. EOD, early-onset dementia; EOAD, early-onset Alzheimer’s disease; EOFTD, early-onset frontotemporal dementia.

The present study represents a retrospective analysis of a prospectively recruited cohort of consecutive patients evaluated at the Memory Clinic, Neurology Clinic, University Clinical Center of Serbia, Belgrade, between April 2012 and April 2017. For the purposes of this study, EOD was defined as symptom onset before 65 years of age. During the study period, 263 consecutive patients referred with suspected degenerative early-onset dementia (EOD) underwent comprehensive diagnostic assessment. Of these, 208 patients met the study inclusion criteria and fulfilled established diagnostic criteria for probable AD or FTD, comprising 130 patients with EOAD and 78 patients with EOFTD. The remaining 55 patients were excluded for the reasons detailed in Figure 1.

The present cohort overlaps with previously published Serbian cohorts. Of the 208 patients included in the present study, 117 were included in a previous study specifically investigating *C9orf72* repeat expansions [18], whereas all 208 were subsequently included in a broader Memory Center cohort investigating *C9orf72*, *GRN*, and *MAPT* across both early- and later-onset cognitive disorders [20]. There was no patient overlap with the earlier Serbian AD cohort reported by Dobričić et al. [19]. The extent of cohort overlap, previously reported genetic findings, and the specific contributions of the present study are summarized in Supplementary Table S1.

All study participants underwent a comprehensive clinical assessment performed by a neurologist specializing in cognitive disorders. Among the 208 included patients, 130 fulfilled the 2011 National Institute on Aging–Alzheimer’s Association (NIA-AA) criteria for probable AD [21]. Based on their predominant clinical presentation, patients with EOAD were classified into amnestic (*n* = 95, 73%) and non-amnestic phenotypes (*n* = 35, 27%).

Seventy-eight patients fulfilled consensus diagnostic criteria for FTD spectrum disorders. Of these, 60 (77%) met the criteria for probable behavioral variant FTD (bvFTD) [22], while 18 (23%) fulfilled the diagnostic criteria for primary progressive aphasia (PPA) [23], including 13 patients with the nonfluent/agrammatic variant (nfvPPA) and five with the semantic variant (svPPA). Four patients with the logopenic variant of PPA (lvPPA) were included in the non-amnestic EOAD group; in three, the diagnosis was supported by cerebrospinal fluid (CSF) biomarker evidence of AD pathology. The remaining patient, for whom CSF biomarkers were unavailable, was classified within the EOAD group based on the clinical phenotype, characterized by predominant language impairment accompanied by episodic memory impairment, together with an [18F] fluorodeoxyglucose positron emission tomography (FDG-PET) pattern showing severe left temporoparietal hypometabolism with involvement of the posterior cingulate cortex. Frontotemporal dementia–motor neuron disease (FTD–MND) overlap was defined by the coexistence of FTD and MND fulfilling the revised El Escorial criteria [24]. Frontotemporal dementia–progressive supranuclear palsy (FTD–PSP) overlap was defined by the coexistence of FTD and PSP fulfilling the National Institute of Neurological Disorders and Stroke–Society for Progressive Supranuclear Palsy (NINDS-SPSP) criteria [25], while frontotemporal dementia–corticobasal syndrome (FTD–CBS) overlap was defined by the coexistence of FTD and CBS according to the Armstrong et al. criteria [26]. Patients were followed clinically at approximately 6-month intervals throughout the course of the disease, generally until death.

Patient recruitment and biospecimen collection began in 2012. Approval for the retrospective use and analysis of clinical data and biological samples collected from 2012 onward, including genetic testing and CSF biomarker analyses, was obtained from the Ethics Committee of the Faculty of Medicine, University of Belgrade, Serbia (Approval Code: 29/XII-3; 28 December 2015). Additional approval for the present retrospective analysis and re-evaluation was obtained from the Ethics Committee of the University Clinical Centre of Serbia (Approval Codes: 705/28 and 705/3; 23 July 2026), in accordance with subsequent institutional requirements. Written informed consent was obtained from all participants prior to study enrollment.

Clinical assessment included a structured interview with patients and caregivers to collect demographic and clinical data, including education, sex, age at symptom onset, family history of dementia and other neurodegenerative or psychiatric disorders, predominant symptoms at disease onset (cognitive, behavioral, or motor), and the evolution of clinical features during the disease course. Patients underwent neurological and neuropsychological evaluation and were followed at six-month intervals. Whenever feasible, a comprehensive neuropsychological assessment was performed, including at least two tests for each major cognitive domain: verbal learning and memory, visual memory, language, attention, executive functions, and visuospatial abilities.

The diagnostic work-up included routine laboratory testing and neuroimaging to exclude potentially reversible or secondary causes of cognitive impairment. Laboratory investigations included serum biochemistry, vitamin B12 levels, thyroid function tests, erythrocyte sedimentation rate, and HIV testing, as clinically indicated. All patients underwent structural neuroimaging with brain magnetic resonance imaging (MRI) at 1.5 T or, when MRI was contraindicated or unavailable, computed tomography (CT). FDG-PET was performed in 95/130 (73.1%) EOAD and 63/78 (80.8%) EOFTD patients.

CSF analysis included routine cytological and biochemical assessment. CSF amyloid-β42 (Aβ42), total tau (t-tau), and phosphorylated tau (p-tau) concentrations were measured using INNOTEST enzyme-linked immunosorbent assays (ELISA; Innogenetics, Ghent, Belgium) and interpreted according to the laboratory-specific cut-off values used during the study period. In the clinical diagnostic assessment, CSF biomarkers were used as supportive rather than independently diagnostic evidence. An Aβ42 concentration below the laboratory-specific cutoff was considered evidence of amyloid abnormality in the appropriate clinical context, while t-tau and p-tau concentrations provided additional biomarker information. CSF biomarker data were available for 83/130 (63.8%) patients with EOAD and 42/78 (53.8%) patients with EOFTD. In patients with EOFTD, CSF biomarker assessment was used as part of the diagnostic work-up and differential diagnosis from AD.

Detailed family history data were obtained through interviews with at least one caregiver and supplemented by available medical records. Information was collected on family history of AD, FTD, motor neuron disease, and psychiatric symptoms associated with dementia. Familial aggregation and patterns of inheritance were classified using the modified Goldman score [27,28]. Each patient was assigned to one of five categories: (1) autosomal dominant inheritance, defined as at least three affected family members across two generations, with one individual being a first-degree relative of the other two; (2) familial aggregation, defined as three or more affected family members with dementia without fulfilling criteria for autosomal dominant inheritance; (3) one first-degree relative with early-onset dementia (symptom onset before 65 years of age); (3.5) one first-degree relative with late-onset dementia (symptom onset at or after 65 years of age); and (4) no known or unavailable family history.

### 2.2. Genetic Testing

Peripheral blood samples were collected from all participants, and DNA was extracted using standard procedures. All patients with EOAD (*n* = 130) underwent genetic testing of selected coding regions of *APP*, *PSEN1*, *MAPT*, and *GRN*, as well as testing for the *C9orf72* hexanucleotide repeat expansion. All patients with EOFTD (*n* = 78) were tested for variants in *MAPT* and *GRN* and for the *C9orf72* hexanucleotide repeat expansion. Additional testing of *VCP* was performed in four genetically unresolved patients with EOFTD and an autosomal dominant family history, whereas *ANG* was analyzed in three genetically unresolved patients with an FTD–MND overlap syndrome. Selected coding regions of *APP* (exons 16 and 17), *PSEN1* (coding exons), *MAPT* (exons 1 and 9–13), and *GRN* (all coding exons), the entire coding region of *ANG*, and exon 5 of *VCP*, together with surrounding intronic regions, were PCR amplified and sequenced using BigDye Terminator v3.1 Cycle Sequencing chemistry on an ABI 3500 Genetic Analyzer (Applied Biosystems, Foster City, CA, USA). Overall, the number of patients tested for each gene or repeat expansion was as follows: *APP*, *n* = 130; *PSEN1*, *n* = 130; *MAPT*, *n* = 208; *GRN*, *n* = 208; *C9orf72*, *n* = 208; *VCP*, *n* = 4; and *ANG*, *n* = 3. *PSEN2* and copy-number variants were not analyzed. APOE genotyping was available for 120 patients with EOAD and 76 patients with EOFTD. The *APOE* genotype was determined by analysis of the rs429358 and rs7412 single-nucleotide polymorphisms using commercial TaqMan SNP Genotyping Assays on an ABI 7500 Real-Time PCR System (Applied Biosystems, Foster City, CA, USA).

Genetic testing of this cohort was performed between 2015 and 2018. At the time of the original genetic analysis, detected variants were checked against the Human Gene Mutation Database (HGMD) and the Alzheimer Disease and Frontotemporal Dementia Mutation Database [29] to determine whether they had been previously reported. The predicted functional effects of previously unreported non-synonymous coding variants were assessed using web-based in silico prediction tools, including MutationTaster2 and PROVEAN v1.1.5 [30,31]. For the present study, identified variants were re-evaluated in 2026 using current publicly available resources, including ClinVar [32], gnomAD [33], and the available published literature. All identified variants were classified according to the American College of Medical Genetics and Genomics/Association for Molecular Pathology (ACMG/AMP) guidelines [34].

The *C9orf72* region containing the hexanucleotide repeat was analyzed using standard quantitative fluorescent PCR (QF-PCR) and repeat-primed PCR (RP-PCR) with previously published primers [35,36]. Allele sizing was performed by capillary electrophoresis on an ABI 3500 Genetic Analyzer (Applied Biosystems, Foster City, CA, USA). Samples showing an expanded RP-PCR pattern were further analyzed by Southern blotting using an end-labeled (GGGGCC)_4_ DIG probe to confirm that the repeat number exceeded the cut-off of 30 repeats used during the study period (for details, see [18]).

### 2.3. Statistical Analysis

Statistical analyses were performed using SPSS version 16.0 (SPSS Inc., Chicago, IL, USA). The distribution of continuous variables was assessed using the Shapiro–Wilk test. Descriptive statistics are presented as proportions for categorical variables and as means and standard deviations (SDs) for continuous variables. Categorical variables were analyzed using the chi-square test or Fisher’s exact test, as appropriate, according to expected cell frequencies. Comparisons of individual modified Goldman score categories were performed using Fisher’s exact test. Continuous variables were compared between two groups using the independent-samples Student’s *t*-test for normally distributed variables and the Mann–Whitney *U* test for non-normally distributed variables. The CDR global score, as an ordinal variable, was compared between groups using the Mann–Whitney *U* test. Comparisons of CSF biomarker concentrations between the EOAD and EOFTD groups were considered descriptive because CSF biomarker findings contributed to the clinical diagnostic assessment. An exploratory subgroup analysis was performed in patients with symptom onset before 51 years of age, using the cutoff applied in a previous genetic study of very early-onset AD [37] to facilitate comparison with published data. Missing data were not imputed; analyses were performed using available data for each variable. No adjustment for multiple comparisons was applied. A two-sided *p* value < 0.05 was considered statistically significant. Exact binomial 95% confidence intervals (CIs) were calculated for diagnostic yield estimates.

## 3. Results

### 3.1. Demographic and Clinical Characteristics

The demographic and clinical characteristics of EOAD and EOFTD patients are summarized in Table 1. No significant differences were observed between the two groups with respect to age, age at symptom onset, disease duration, sex distribution, or years of education. CSF biomarker data were available in 125 patients and were used as supportive evidence within the clinical diagnostic assessment. Descriptively, patients with EOAD had lower CSF Aβ42 concentrations and higher total tau and phosphorylated tau concentrations than patients with EOFTD (Table 1).

### 3.2. Clinical Phenotypes

**Figure 2:**
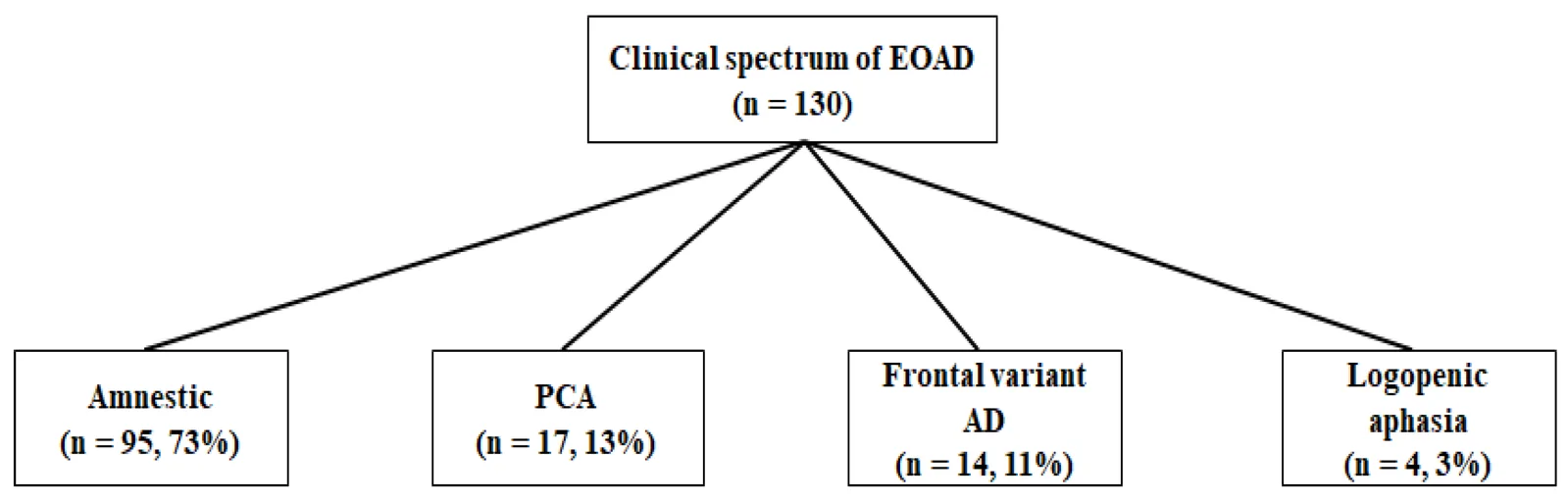
Distribution of clinical phenotypes in early-onset Alzheimer’s disease (EOAD). **Abbreviations:** AD, Alzheimer’s disease; EOAD, early-onset Alzheimer’s disease; PCA, posterior cortical atrophy.

Among the 130 patients with EOAD, the amnestic phenotype was the most common, occurring in 95 patients (73%). Non-amnestic presentations were observed in 35 patients (27%), including posterior cortical atrophy (PCA; *n* = 17, 13%), frontal variant AD (*n* = 14, 11%), and lvPPA (*n* = 4, 3%) (Figure 2).

**Figure 3:**
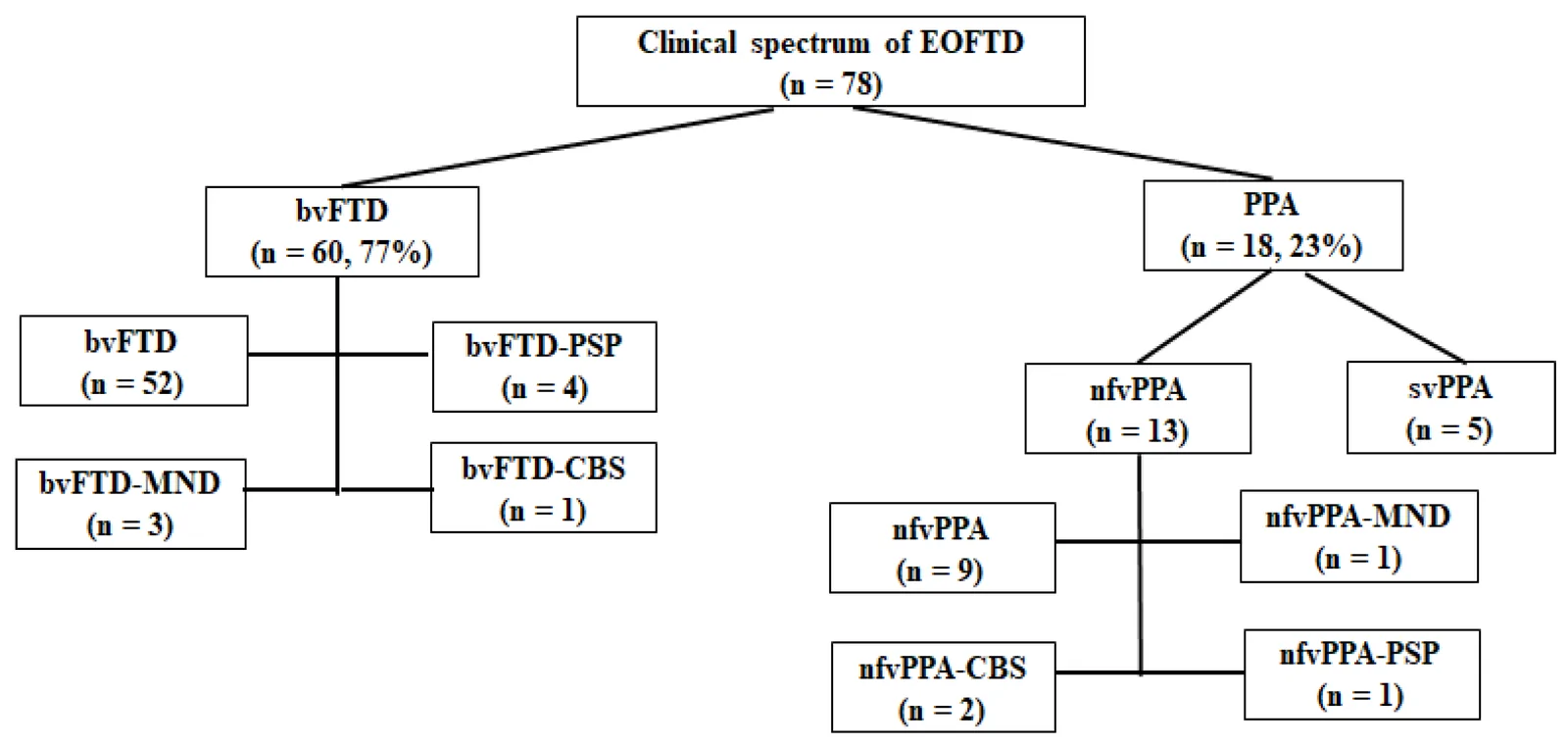
Distribution of clinical phenotypes and overlap syndromes in early-onset frontotemporal dementia (EOFTD). **Abbreviations:** bvFTD, behavioral variant frontotemporal dementia; CBS, corticobasal syndrome; EOFTD, early-onset frontotemporal dementia; MND, motor neuron disease; PPA, primary progressive aphasia; nfvPPA, nonfluent/agrammatic variant PPA; PSP, progressive supranuclear palsy; svPPA, semantic variant primary progressive aphasia.

Among the 78 patients with EOFTD, behavioral variant FTD (bvFTD) was the predominant clinical phenotype (*n* = 60, 77%). Of these, eight patients developed clinical overlap syndromes during the disease course, including FTD–PSP (*n* = 4), FTD–MND (*n* = 3), and FTD–CBS (*n* = 1). The remaining 18 patients (23%) presented with primary progressive aphasia (PPA), comprising nonfluent/agrammatic variant PPA (nfvPPA; *n* = 13) and semantic variant PPA (svPPA; *n* = 5). Among patients with nfvPPA, overlap syndromes included nfvPPA-CBS (*n* = 2), nfvPPA-PSP (*n* = 1), and nfvPPA-MND (*n* = 1) (Figure 3).

### 3.3. Family History

According to the modified Goldman score, 42 patients with EOAD (32.3%) and 24 patients with EOFTD (30.8%) had a positive family history of dementia (Goldman scores 1–3.5), whereas the majority of patients in both groups had a modified Goldman score of 4, indicating no known or unavailable family history (Table 2). Only 7 patients with EOAD (5.4%) and 6 with EOFTD (7.7%) fulfilled the criteria for autosomal dominant inheritance (Goldman score 1). The distribution of modified Goldman scores was similar between the two groups. In an exploratory category-specific comparison, Goldman score 3.5 was more common in patients with EOAD than in those with EOFTD (20.0% vs. 7.7%, *p* = 0.018) (Table 2).

The distribution of modified Goldman scores according to the clinical phenotypes of EOAD and EOFTD is presented in Supplementary Tables S2 and S3.

### 3.4. Genetic Findings

#### 3.4.1. EOAD Cohort

Pathogenic or likely pathogenic variants were identified in 5 of 130 patients with EOAD (3.8%, 95% CI, 1.3–8.7%).

In the exploratory subgroup analysis of patients with symptom onset before 51 years of age, pathogenic or likely pathogenic variants were identified in 4 of 12 patients (33.3%; 95% CI, 9.9–65.1%).

Four previously reported pathogenic variants were identified: two in *PSEN1* (p.Met139Thr, c.416T > C; p.Arg269His, c.806G > A) and two in *APP* (p.Thr714Ile, c.2141C > T; p.Val717Ile, c.2149G > A). The remaining variant, the previously reported *PSEN1* p.Tyr159Cys (c.476A > G), which had originally been considered potentially pathogenic based on in silico predictions, is currently classified as likely pathogenic according to ACMG/AMP criteria (Table 3).

No pathogenic variants in *MAPT* or *GRN*, or pathogenic *C9orf72* repeat expansions, were identified in the EOAD cohort.

APOE genotyping was available for 120 patients with EOAD. The *APOE* ε3/ε4 and ε4/ε4 genotypes were present in 44 (36.7%) and 7 (5.8%) patients, respectively. Among the five carriers of pathogenic or likely pathogenic variants, three had an ε3/ε4 genotype and two had an ε3/ε3 genotype.

Among the 42 patients with a positive family history of dementia (modified Goldman scores 1–3.5), four (9.5%; 95% CI, 2.7–22.6%) carried a pathogenic or likely pathogenic variant. Of the five variant carriers overall, three had a modified Goldman score of 1, consistent with autosomal dominant inheritance, one had a Goldman score of 3, and one had a modified Goldman score of 4 (no known or unavailable family history) (Table 3).

Patients carrying pathogenic or likely pathogenic variants had a significantly younger age at symptom onset than non-carriers (48.0 ± 6.4 vs. 57.2 ± 4.4 years, *p* < 0.01).

Among the five variant carriers, four presented with the amnestic phenotype, whereas one carrying a *PSEN1* variant presented with posterior cortical atrophy (Table 3).

Detailed current variant classifications, RefSeq transcripts, HGVS nomenclature, gnomAD population frequencies, and ACMG/AMP evidence codes are provided in Supplementary Table S4.

Individual CSF biomarker values (pg/mL), when available, were as follows: *APP* p.Val717Ile, Aβ42 368.59, t-tau 250.93, p-tau 51.90; *PSEN1* p.Met139Thr, Aβ42 435.72, t-tau 825.52, p-tau 91.64; *PSEN1* p.Tyr159Cys, Aβ42 282.09, t-tau 336.53, p-tau 58.80. CSF data were not available for the remaining two variant carriers.

#### 3.4.2. EOFTD Cohort

Pathogenic or likely pathogenic variants or a pathogenic repeat expansion were identified in 7 of 78 patients with EOFTD (9.0%; 95% CI, 3.7–17.6%). Six patients (7.7%) carried pathogenic variants or a pathogenic repeat expansion in the major FTD-associated genes, including five with a pathogenic *C9orf72* hexanucleotide repeat expansion and one with the previously reported pathogenic *GRN* variant (p.Arg418Ter, c.1252C > T). In addition, one patient carried the previously reported pathogenic *VCP* p.Gly157Arg (c.469G > A) variant, representing a rare genetic cause of FTD. The *ANG* p.Met1Ile (c.3G > A) variant identified in another patient is currently classified as a variant of uncertain significance (VUS) and was therefore not included in the diagnostic yield (Table 4).

No pathogenic variants were identified in the *MAPT* gene in the EOFTD cohort.

APOE genotyping was available for 76 patients with EOFTD. The *APOE* ε3/ε4 and ε4/ε4 genotypes were present in 10 (13.2%) and 1 (1.3%) patients, respectively. Among the seven carriers of pathogenic or likely pathogenic variants or pathogenic repeat expansion, five had an ε3/ε3 genotype and two had an ε2/ε3 genotype.

Among the 24 patients with a positive family history (modified Goldman scores 1–3.5), six (25.0%;95% CI, 9.8–46.7%) carried a pathogenic or likely pathogenic variant or pathogenic repeat expansion. Of the seven patients with a pathogenic or likely pathogenic variant or pathogenic repeat expansion, three had a modified Goldman score of 1, consistent with autosomal dominant inheritance, three had a Goldman score of 3, and one had a Goldman score of 4 (no known or unavailable family history) (Table 4). Four of the five patients carrying a *C9orf72* repeat expansion had a positive family history; however, none had a family history of MND. All affected relatives had EOD.

Patients carrying a pathogenic or likely pathogenic variant or pathogenic repeat expansion had a younger mean age at symptom onset than non-carriers (53.7 ± 8.1 vs. 56.2 ± 7.1 years), although the difference was not statistically significant (*p* = 0.39). Six of the seven patients with a pathogenic or likely pathogenic variant or pathogenic repeat expansion presented with bvFTD or bvFTD–motor neuron disease overlap, whereas the *GRN* variant carrier presented with nfvPPA (Table 4).

Detailed current variant classifications, RefSeq transcripts, HGVS nomenclature, gnomAD population frequencies, and ACMG/AMP evidence codes are provided in Supplementary Table S4.

Individual CSF biomarker values (pg/mL), when available, were as follows: *GRN* p.Arg418Ter, Aβ42 1052.32, t-tau 421.82, p-tau 43.66; *ANG* p.Met1Ile, Aβ42 967.69, t-tau 367.10, p-tau 37.09; *C9orf72* repeat expansion carriers: onset age 50 years, Aβ42 993.15, t-tau 215.11, p-tau 45.46; onset age 52 years, Aβ42 734.10, t-tau 314.51, p-tau 34.22. CSF data were not available for the remaining four variant carriers.

## 4. Discussion

In this study, we characterized the genetic spectrum, diagnostic yield of genetic testing, and family history in a consecutive cohort of 208 Serbian patients with EOD, including EOAD and EOFTD, evaluated at a single tertiary memory clinic.

Approximately one-third of patients had a positive family history of dementia or another neurodegenerative disorder. Pathogenic or likely pathogenic variants or pathogenic repeat expansions were identified in 3.8% of patients with EOAD and 9.0% of those with EOFTD. In an exploratory subgroup analysis, a higher diagnostic yield was observed among patients with very early-onset EOAD, while a positive family history was associated with a higher frequency of pathogenic or likely pathogenic variants or pathogenic repeat expansions in both cohorts. *PSEN1* and *APP* were the only genes harboring pathogenic or likely pathogenic variants in EOAD, whereas *C9orf72* repeat expansions were the predominant genetic finding in EOFTD, followed by *GRN* and *VCP* variants. Collectively, these findings demonstrate that the molecular diagnostic yield of targeted genetic testing varies according to clinical phenotype, age at onset, and family history in patients with EOD. However, the substantial proportion of genetically unresolved familial cases may reflect incomplete assessment of known genetic causes, including genes and variant classes not covered by the targeted testing strategy used in this study, and supports the use of more comprehensive genomic approaches in future studies.

Analysis of family history revealed that approximately one-third of patients in both the EOAD and EOFTD cohorts had a positive family history of dementia or another neurodegenerative disorder. Previous European studies reported similar frequencies of familial disease, including 32% among 231 unrelated patients with EOAD from nine Iberian centers [38]. Likewise, a German cohort of 121 consecutive patients with FTD across the age spectrum reported a positive family history in 33% of patients [39]. Consistent results were observed in an Australian cohort, in which a positive family history was present in 27.5% of patients with EOAD and 25% of those with EOFTD [17]. The concordance across these studies likely reflects similar study designs based on consecutive inclusion of patients irrespective of family history, thereby minimizing referral bias. Some variation across cohorts is nevertheless evident. A lower frequency of positive family history (23%) was reported in a Korean cohort of 104 consecutively recruited patients with EOAD [40]. The authors attributed this finding primarily to the unbiased nationwide recruitment strategy, although they also proposed an earlier onset of sporadic EOAD in the Korean population as a possible explanation. In contrast, studies employing selection criteria that enriched for familial or young-onset cases consistently reported substantially higher proportions of familial disease, including 73% in a Spanish EOAD series and 40.1% in an FTD cohort [41,42]. Likewise, our previous Serbian EOAD study, which specifically recruited patients with familial and/or young-onset AD, reported familial disease in 61.7% of cases [19], underscoring the influence of cohort selection on estimates of familial aggregation in EOD.

Among patients with a positive family history, the pattern of inheritance ranged from autosomal dominant pedigrees to families with only a single affected first-degree relative. Autosomal dominant inheritance (Goldman score 1) was observed in only 5.4% of patients with EOAD and 7.7% of those with EOFTD, consistent with previous studies showing that only a minority of early-onset degenerative dementias follow an autosomal dominant pattern, accounting for approximately 10–15% of cases [17,27,42,43,44,45,46]. Thus, while a positive family history is common in EOD, only a small proportion of patients exhibit an autosomal dominant pattern of inheritance.

Although the proportion of patients with a single affected first-degree relative before the age of 65 was similar between the EOAD and EOFTD cohorts, a first-degree relative with late-onset dementia (symptom onset at or after 65 years of age) was significantly more common in EOAD (20% vs. 7.7%). This observation supports the concept that EOAD and LOAD share genetic susceptibility, with both rare, highly penetrant variants and additional genetic modifiers contributing to the age at disease onset [47]. These families may represent an important resource for identifying additional genetic risk factors for AD.

The overall diagnostic yield of genetic testing in our EOAD cohort was 3.8%, increasing to 9.5% among patients with a positive family history. These findings are comparable to those reported by Guerreiro et al., who identified pathogenic variants in 3.9% of an unselected EOAD cohort and in 8.1% of familial cases [38]. Similarly, Jarmolowicz et al. reported a diagnostic yield of 1.6% in a consecutive EOAD cohort, although genetic testing in that study was restricted to patients with an autosomal dominant family history [17]. Notably, pathogenic or likely pathogenic variants were identified in only three of seven (43%) autosomal dominant EOAD families in our cohort, leaving more than half of these pedigrees genetically unresolved. This is consistent with previous studies, in which the proportion of genetically resolved autosomal dominant EOAD families varied considerably across cohorts [17,41,48,49]. The substantial proportion of genetically unresolved families may, at least in part, reflect the incomplete assessment of known genetic causes by the targeted testing strategy used in this study. Despite advances in sequencing technologies and the expanding spectrum of EOAD-associated genes, many families with apparent autosomal dominant inheritance still lack an identifiable molecular diagnosis [7,50].

An exploratory finding of our study was the higher diagnostic yield observed among patients with disease onset before 51 years of age. This cutoff was selected to facilitate comparison with a previous genetic study of very early-onset AD using the same age threshold [37]. Pathogenic or likely pathogenic variants were identified in four of 12 patients (33.3%) in this subgroup. This finding is consistent with previous studies showing that very early symptom onset is associated with an increased likelihood of identifying highly penetrant pathogenic variants in autosomal dominant AD genes, including among apparently sporadic cases [37,51]. Notably, one patient without a family history carried the likely pathogenic *PSEN1* p.Tyr159Cys variant. The early age at onset (49 years), a CSF biomarker profile consistent with AD, and the occurrence of early myoclonic seizures further support the pathogenicity of this variant. Because parental DNA was unavailable, the possibility of a de novo origin could not be confirmed and remains speculative. Previous genetic screening studies have shown that a substantial proportion of *PSEN1* mutations in apparently sporadic EOAD arise de novo and that pathogenic *APP/PSEN1* variants are detected in approximately 12–13% of apparently sporadic EOAD cases with disease onset before 51 years [37,51].

Four of the five genetically confirmed EOAD patients in our cohort presented with the typical amnestic phenotype, whereas one patient carrying a pathogenic *PSEN1* variant had a clinical presentation consistent with posterior cortical atrophy. This observation illustrates the marked phenotypic heterogeneity associated with *PSEN1* variants. Although amnestic Alzheimer’s disease remains the predominant presentation of autosomal dominant EOAD, atypical cognitive syndromes occur more frequently in *PSEN1* than in *APP* variant carriers, and both age at onset and clinical phenotype are influenced by the underlying causative variant [52,53].

Pathogenic variants in *MAPT*, *GRN*, and *C9orf72* have occasionally been identified in patients with a clinical diagnosis of Alzheimer’s disease. However, no pathogenic variants in these genes were detected in our EOAD cohort, consistent with current evidence indicating that they are uncommon causes of clinically diagnosed EOAD in the absence of prominent frontotemporal features [50,54].

The overall diagnostic yield of genetic testing in our EOFTD cohort was 9.0%, increasing to 25.0% among patients with a positive family history, in whom pathogenic or likely pathogenic variants or pathogenic repeat expansions were identified in six of 24 cases. Pathogenic or likely pathogenic variants or pathogenic repeat expansions were also identified in three of six (50%) autosomal dominant EOFTD families. These findings are consistent with those reported by Jarmolowicz et al., who identified pathogenic variants in 7.3% of an unselected EOFTD cohort and in 45.5% of autosomal dominant EOFTD families [17].

Large FTD cohorts across the age spectrum have similarly identified *C9orf72* repeat expansion as the most common genetic cause [55,56]. In our cohort, it accounted for five of the seven genetically confirmed cases. This distribution was also observed in a recent analysis from the Belgrade Memory Center, where *C9orf72* repeat expansion was the most frequent genetic cause among patients with FTD [20]. In addition, pathogenic variants in *GRN* and *VCP* further illustrated the marked genetic heterogeneity of EOFTD. Notably, *VCP* is a rare genetic cause of FTD. The *ANG* p.Met1Ile variant identified in our cohort is currently classified as a VUS and was therefore not considered a diagnostic genetic finding, although *ANG* variants have been reported predominantly in motor neuron disease, with occasional descriptions in patients with overlapping FTD–MND phenotypes [57,58,59]. Further illustrating the contribution of genetic testing to molecular characterization, an additional patient initially diagnosed with an FTD–PSP overlap syndrome was subsequently found to have adult-onset Niemann–Pick disease type C caused by biallelic *NPC1* variants and was therefore excluded from the present cohort [60]. Although not included in the study cohort, this case emphasizes the importance of considering alternative inherited neurodegenerative disorders in patients with atypical neurological or systemic features and, together with previous reports of adult-onset lysosomal storage disorders presenting with FTD-like phenotypes [39,61], underscores the expanding spectrum of inherited disorders that may clinically mimic FTD.

A pathogenic genetic finding was identified in 1 of 54 (1.9%) EOFTD patients with a modified Goldman score of 4, consisting of a *C9orf72* repeat expansion. This patient presented with a bvFTD–MND overlap phenotype. Although the diagnostic yield in this subgroup was low, this finding illustrates that a pathogenic genetic alteration may be identified even in patients without a clearly documented positive family history. Such presentations may reflect reduced or age-dependent penetrance, limited family structure, or unrecognized disease in previous generations, all of which have been described in FTD-associated genes [39]. The identification of a *C9orf72* repeat expansion in a patient with bvFTD–MND is consistent with previous studies showing that *C9orf72* repeat expansion is the most common genetic cause of FTD–MND [62]. It also aligns with recent recommendations supporting the integration of genetic counseling and comprehensive genetic testing into the routine clinical care of patients with FTD, regardless of family history [63].

Consistent with previous studies [42,59], bvFTD and bvFTD–MND overlap syndrome were the predominant clinical presentations among genetically confirmed EOFTD patients, accounting for six of seven cases (85.7%). *C9orf72* repeat expansion was the most frequent genetic cause, whereas a *VCP* pathogenic variant was also associated with bvFTD. The only non-bvFTD presentation occurred in the patient carrying a *GRN* pathogenic variant, who presented with nfvPPA. Overall, our findings are consistent with the recognized genotype–phenotype spectrum of hereditary FTD and further illustrate its marked clinical heterogeneity.

This study has several limitations. First, the diagnosis was based on clinical criteria without neuropathological confirmation, which may have resulted in some degree of diagnostic misclassification, particularly given the clinical overlap between neurodegenerative disorders. However, CSF biomarkers and/or FDG-PET were available in the majority of patients and provided additional support for the clinical diagnoses. The Aβ42/Aβ40 ratio was not available during the study period, which represents a limitation of the CSF biomarker assessment. Second, family history information was partly based on caregiver reports because medical documentation was unavailable for some affected relatives. In addition, modified Goldman score 4 included both patients with no known family history and those for whom family history information was unavailable. Consequently, patients in this category cannot uniformly be considered to have sporadic disease, and family history may have been underestimated or incompletely characterized. Third, genetic testing was based on a phenotype-directed candidate-gene approach rather than comprehensive next-generation sequencing. The scope of testing was therefore not uniform across all patients, particularly within the EOFTD cohort, and Sanger sequencing was restricted to the selected gene regions specified in the Methods; copy-number variant analysis was not performed. Consequently, pathogenic variants outside the analyzed regions, in genes not included in the testing strategy, or involving copy-number changes may have been missed, potentially leading to an underestimation of the diagnostic yield and limiting direct comparisons with studies using broader NGS panels, whole-exome sequencing, or whole-genome sequencing. Finally, the relatively small number of genetically confirmed cases limited detailed genotype–phenotype analyses and the interpretation of findings within specific genetic and clinical subgroups. In particular, estimates derived from small subgroups, including the diagnostic yield observed among patients with symptom onset before 51 years of age, should be interpreted cautiously and require confirmation in larger cohorts. As this was a single-center study, the findings may not be fully generalizable to other populations or clinical settings, and multicenter studies are warranted to validate these observations in broader populations.

## 5. Conclusions

In conclusion, *PSEN1* and *APP* were the only disease-causing genes identified in EOAD, whereas *C9orf72* repeat expansion was the predominant genetic cause of EOFTD, with additional pathogenic variants identified in *GRN* and *VCP*. The diagnostic yield of targeted genetic testing was higher among patients with a positive family history and, in an exploratory analysis, among patients with very early disease onset, indicating that these clinical features may help identify patients more likely to have a detectable pathogenic genetic finding. Nevertheless, the substantial proportion of genetically unexplained autosomal dominant families may, at least in part, reflect incomplete assessment of known genetic causes by the targeted testing strategy used in this study, supporting the use of more comprehensive genomic approaches to improve molecular diagnostic yield. From a broader diagnostic perspective, the evaluation of EOD should integrate detailed clinical and neuropsychological assessment with neuroimaging, disease-specific biomarkers, and appropriately targeted genetic testing.

## Figures and Tables

**Table 1:** Demographic and clinical characteristics of EOAD and EOFTD patients.

	EOAD	EOFTD	*p*
*n*	130	78	
Age (years)	60.3 ± 5.0 (38–69)	59.8 ± 6.8 (39–70)	0.51
Age at onset (years)	56.8 ± 4.7 (37–64)	56.0 ± 7.2 (31–64)	0.29
Disease duration (years)	3.5 ± 1.5 (1–7)	3.8 ± 2.4 (1–13)	0.26
Education (years)	11.5 ± 2.9 (4–18)	12.1 ± 3.1 (0–16)	0.17
Female sex (%)	78 (60%)	38 (49%)	0.11
CDR-GS	2.0 (1.0–3.0)	1.0 (1.0–2.0)	<0.001
MMSE	14.1 ± 6.5	18.1 ± 9.6	<0.001
CSFAβ42 * (*n* = 83/42)	416.5 ± 154.0	975.6 ± 165.4	<0.001
CSF t-tau * (*n* = 83/42)	611.36 ± 284.4	310.6 ± 165.4	<0.001
CSF p-tau * (*n* = 83/42)	92.7 ± 38.0	56.63 ± 34.2	<0.001

Data are presented as mean ± standard deviation (range), median (interquartile range [IQR]), or n (%), as appropriate. *p* values were calculated using the independent-samples *t* test for continuous variables, the Mann–Whitney *U* test for CDR-GS and the χ^2^ test for categorical variables. MMSE data were available for 130 patients with EOAD and 76 patients with EOFTD. **Abbreviations:** Aβ42, amyloid-β42; CDR-GS, Clinical Dementia Rating global score; CSF, cerebrospinal fluid; EOAD, early-onset Alzheimer’s disease; EOFTD, early-onset frontotemporal dementia; MMSE, Mini-Mental State Examination; p-tau, phosphorylated tau; t-tau, total tau. * CSF biomarker data were available for 83/130 (63.8%) patients with EOAD and 42/78 (53.8%) patients with EOFTD. The laboratory cutoff values were Aβ42 < 563.1 ng/L, t-tau > 244.7 ng/L, and p-tau > 83.4 ng/L. Values below the cutoff for Aβ42 and above the cutoffs for t-tau and p-tau were considered abnormal.

**Table 2:** Family history according to the modified Goldman score in patients with EOAD and EOFTD.

	EOAD	EOFTD	*p*
*n*	130	78	
Modified Goldman score			
1	7 (5.4)	6 (7.7)	NS
2	0	0	
3	9 (6.9)	12 (15.4)	NS
3.5	26 (20)	6 (7.7)	=0.018
**4**	**88 (67.7)**	**54 (69.2)**	**NS**
**Positive family history** **(Goldman scores 1–3.5)**	**42 (32.3)**	**24 (30.8)**	**NS**

Data are presented as *n* (%). Category-specific *p* values were calculated using Fisher’s exact test and were considered exploratory; no adjustment for multiple comparisons was applied. NS, not significant. Family history was defined as a modified Goldman score of 1–3.5. Bold indicates the two summary categories: modified Goldman score 4 and positive family history (modified Goldman scores 1–3.5).

**Table 3:** Clinical and genetic characteristics of patients with EOAD carrying pathogenic or likely pathogenic variants.

Gene	Protein Change	Nucleotide Change	Exon	Age at Onset (Years)	Sex	Clinical Phenotype	Modified Goldman Score
*APP*	p.Thr714Ile	c.2141C > T	17	37	F	Amnestic	1
*APP*	p.Val717Ile	c.2149G > A	17	50	F	Amnestic	1
*PSEN1*	p.Arg269His	c.806G > A	8	54	M	Posterior cortical atrophy	1
*PSEN1*	p.Met139Thr	c.416T > C	5	50	F	Amnestic	3
*PSEN1*	p.Tyr159Cys †	c.476A > G	5	49	F	Amnestic	4

† Initially considered potentially pathogenic based on in silico prediction and currently classified as likely pathogenic according to ACMG/AMP criteria. **Abbreviations:** EOAD, early-onset Alzheimer’s disease.

**Table 4:** Clinical and genetic characteristics of patients with EOFTD carrying pathogenic or likely pathogenic variants, pathogenic repeat expansions, or a variant of uncertain significance.

Gene	Variant	Age at Onset (Years)	Sex	Clinical Phenotype	Modified Goldman Score
*GRN*	p.Arg418Ter (c.1252C > T)	64	F	nfvPPA	1
*VCP*	p.Gly157Arg (c.469G > A)	55	F	bvFTD	1
*ANG*	p.Met1Ile † (c.3G > A)	47	F	bvFTD–MND	4
*C9orf72*	Hexanucleotide repeat expansion	42	F	bvFTD–MND	4
*C9orf72*	Hexanucleotide repeat expansion	49	F	bvFTD	1
*C9orf72*	Hexanucleotide repeat expansion	50	F	bvFTD	3
*C9orf72*	Hexanucleotide repeat expansion	52	F	bvFTD	3
*C9orf72*	Hexanucleotide repeat expansion	64	F	bvFTD	3

† The *ANG* p.Met1Ile variant is currently classified as a variant of uncertain significance (VUS) and was not included in the diagnostic yield. **Abbreviations:** bvFTD, behavioral variant frontotemporal dementia; EOFTD, early-onset frontotemporal dementia; MND, motor neuron disease; nfvPPA, nonfluent/agrammatic variant PPA.

## Data Availability

The raw data supporting the conclusions of this article will be made available by the authors on request.

## Supplementary Materials

The following supporting information can be downloaded at: https://www.mdpi.com/article/10.3390/diseases14100359/s1. Supplementary Table S1: Overlap of the present cohort with previous Serbian studies and new contributions of the present study; Supplementary Table S2: Family history according to the modified Goldman score by clinical phenotype in patients with early-onset Alzheimer’s disease (EOAD); Supplementary Table S3: Family history according to the modified Goldman score by clinical phenotype in patients with early-onset frontotemporal dementia (EOFTD); Supplementary Table S4: Current classification of genetic variants identified in the present cohort.
