# Supplementary material for: Genetic Spectrum, Family History and Diagnostic Yield of Targeted Genetic Testing in Early-Onset Alzheimer’s Disease and Frontotemporal Dementia: A Serbian Memory Clinic
Source: Diseases. 2026 Sep 29;14(10):359. doi: 10.3390/diseases14100359 (PMC13703555; doi:10.3390/diseases14100359)
Supplement: Supplementary file 1 [file diseases-14-00359-s001.zip › diseases-4511326-supplementary.pdf]

Supplementary Table S1. Overlap of the present cohort with previous Serbian studies and new contributions of the present study.

Previous publication	Patients overlapping with the present cohort, n	Genetic findings from the present cohort previously reported	Scope of the previous publication	New contribution of the present study
Dobričić et al., 2012 [18]	0	None	Genetic screening of an independent Serbian cohort with familial and/or early-onset AD	Focused analysis of a consecutively recruited cohort restricted to early-onset AD and FTD, with assessment of diagnostic yield according to family history and clinical characteristics, including age at onset.
Mandić-Stojmenović et al., 2015 [17]	117	Four *C9orf72* repeat expansions	Screening specifically for *C9orf72* repeat expansion in patients with EOD	Extended phenotype-directed genetic testing and evaluation of diagnostic yield in EOAD and EOFTD according to family history and clinical characteristics, including age at onset.
Stefanova et al., 2024 [19]	208	Five *C9orf72* repeat expansions and *GRN* p.Arg418Ter	Analysis of *C9orf72*, *GRN*, and *MAPT* in a broader Memory Center cohort including both early- and later-onset patients with FTD, AD, MCI, and unspecified dementia	Focused analysis of a consecutive EOAD/EOFTD cohort, including additional testing of *APP* and *PSEN1* in EOAD and *VCP* and *ANG* in selected EOFTD patients, with assessment of diagnostic yield according to family history, age at onset, and clinical phenotype

Supplementary Table S2. Family history according to the modified Goldman score by clinical phenotype in patients with early-onset Alzheimer's disease (EOAD).

	**Amnestic**	**PCA**	**Frontal variant AD**	**Logopenic aphasia**
N	95	17	14	4
Modified Goldman score				
1	6(6.3)	1 (5.8)	0	0
2	0	0	0	0
3	7(7.4)	2(11.8)	0	0
3.5	20 (21.1)	1 (5.8)	5(35.7)	0
4	62 (65.2)	13(76.6)	9(64.3)	4
Positive family history (Goldman scores 1-3.5)	33(34.8)	4 (23.4)	5(35.7)	0

Data are presented as n (%).

Supplementary Table S3. Family history according to the modified Goldman score by clinical phenotype in patients with early-onset frontotemporal dementia (EOFTD).

	**bvFTD**	**nfvPPA**	**svPPA**
N	60	13	5
Modified Goldman score			
1#	5(8.3)	1(7.7)	0
2#	0	0	0
3#	12(20)	0	0
3.5#	5(8.3)	1(7.7)	0
4#	38(63.3)	11(84.6)	5(100)
Positive family history (Goldman scores 1-3.5)	22(36.7)	2 (15.4)	0

Data are presented as n (%).

Supplementary Table S4. Current classification of genetic variants identified in the present cohort.

Gene	RefSeq transcript	HGVS nucleotide change	Protein change	gnomAD allele frequency	Current ClinVar classification	ClinVar last evaluated	ACMG/AMP classification	ACMG/AMP evidence codes
APP	NM_000484.4	c.2141C>T	p.Thr714Ile	Not detected	Pathogenic	25 Apr 2024	Pathogenic	PM1, PM2, PP2, PP3, PS4, PS3
APP	NM_000484.4	c.2149G>A	p.Val717Ile	0.000001239	Pathogenic	28 Nov 2025	Pathogenic	PM1, PP2, PP3, PS4, PM2
PSEN1	NM_000021.4	c.806G>A	p.Arg269His	0.000009303	Pathogenic	27 Jan 2026	Pathogenic	PM1, PP2. PS4, PM2, PP3
PSEN1	NM_000021.4	c.416T>C	p.Met139Thr	Not detected	Pathogenic	1 Jul 2020	Pathogenic	PS1, PP1, PP2, PM1, PS4, PM2, PP3
PSEN1	NM_000021.4	c.476A>G	p.Tyr159Cys	0.000001239	No corresponding ClinVar record identified	—	Likely pathogenic	PM1, PM5, PP2, PP3
GRN	NM_002087.4	c.1252C>T	p.Arg418Ter	0.000004338	Pathogenic	24 Oct 2024	Pathogenic	PVS1, PM2, PP3
VCP	NM_007126.5	c.469G>A	p.Gly157Arg	Not detected	Pathogenic	1 Aug 2024	Pathogenic	PM1, PP3, PP2, PS3, PS4, PM2
ANG	NM_001097577.3	c.3G>A	p.Met1Ile	0.0001631	Uncertain significance	15 Feb 2024	VUS	PM2, PP3, PP2
C9orf72	Not applicable	GGGGCC hexanucleotide repeat expansion	Not applicable	Not applicable	Not applicable	—	Not applicable (repeat expansion)	Not applicable (repeat expansion)

Note: The “ACMG/AMP classification” represents the authors’ assessment according to ACMG/AMP criteria, whereas the “Current ClinVar classification” reports the classification available in ClinVar at the time of re-evaluation.
